# Selections from Surgical Notes

**Published:** 1867-08

**Authors:** Moses Gunn

**Affiliations:** Prof. Surgery, Rush Medical College


### CHICAGO MEDICAL JOURNAL.

VOL. XXIV. AUGUST, 1867. No. 8.

### Original Contributions.

### SELECTIONS FROM SURGICAL NOTES.

BY MOSES GUNN, M.D., Prof. Surgery, Rush Medical College.

I select the following two cases of vesical calculus, the first because of the resemblance of the symptoms to neuralgia, and the robust condition of the patient; the second from the fact that a somewhat novel accident complicated the operation.

L. K., a robust German, about 48 years of age, a butcher by occupation, desired my opinion of his case, in consultation with his physician, on the 22d of October, 1865. His height was was about 5 ft. 10 in., weight 240 lbs., complexion florid. He was subject to paroxysms of most excruciating pain referred to penis and scrotum, but more particularly to the glans penis. These paroxysms, which endured from two days to a week, more or less continuously, were greatly aggravated, not only by the act of urination, but also upon the slightest touch upon the genitals; even pointing the finger to the organs would not unfrequently produce painful suffering. At first the general condition of the patient and the character of the pain had led to a diagnosis of neuralgia, and medication had been directed thereto. But as no relief had been obtained after several weeks trial of this class of remedies, suspicions of the presence of a calculus were aroused; a consultation was held, and an unsuccessful attempt to introduce a sound was made. At this stage of the case I was summoned, and so strongly did its physiognomy resemble neuralgia that I had little expectation of finding a stone. I, however, put the patient under the influence of chloroform, and introduced the sound, which soon came in contact with a dense body, giving a very audible click. The lateral operation was made, and presented nothing peculiar except the depth of the perinæral incision rendered necessary by the amount of fat deposited. The calculus was of the uric variety. A good recovery followed.

J. C., aged 51, presented himself at the College Clinic on the 19th of March of the present year. He was greatly emaciated, and experienced all the subjective symptoms of vesical calculus. He had first felt them twenty-seven years previously. On exploration a stricture was discovered, which prevented the introduction of anything larger than a No. 1 sound, by which a large calculus was found in the bladder, and one or more smaller, in the urethra posterior to the stricture. After due preparation of the patient, he was anæthetized, a No. 2 staff introduced, and the operation commenced. After the incision had been made upon the staff, and while the assistant in charge of it attempted to depress it to facilitate opening the urethra, the instrument broke completely off at the commencement of the groove, and I felt the detached portion recede from my touch into the bladder! As I had only with difficulty succeeded in introducing a No. 2 staff, I immediately anticipated the necessity of cutting my way into the bladder on a No. 1 sound. But before doing so I essayed the introduction of a No. 4 staff, and by the use of more force than I should have felt warranted in exercising under less urgent circumstances, I succeeded in the attempt. The incision was completed, and, first, the fragment of the staff was removed; then a large calculus, and two smaller. The large one was an ammonio-magnesian phosphate. The patient made a good recovery.

